# Cellular surface plasmon resonance-based detection of anti-HPA-1a antibody glycosylation in fetal and neonatal alloimmune thrombocytopenia

**DOI:** 10.3389/fimmu.2023.1225603

**Published:** 2023-10-05

**Authors:** Zoltán Szittner, Arthur E. H. Bentlage, A. Robin Temming, David E. Schmidt, Remco Visser, Suzanne Lissenberg-Thunnissen, Juk Yee Mok, Wim J. E. van Esch, Myrthe E. Sonneveld, Erik L. de Graaf, Manfred Wuhrer, Leendert Porcelijn, Masja de Haas, C. Ellen van der Schoot, Gestur Vidarsson

**Affiliations:** ^1^ Immunoglobulin Research Laboratory, Department of Experimental Immunohematology, Sanquin Research, Amsterdam, Netherlands; ^2^ Landsteiner Laboratory Amsterdam University Medical Center, University of Amsterdam, Amsterdam, Netherlands; ^3^ Department of Biomolecular Mass Spectrometry and Proteomics, Utrecht Institute for Pharmaceutical Sciences and Bijvoet Center for Biomolecular Research, Utrecht University, Utrecht, Netherlands; ^4^ Sanquin Reagents, Amsterdam, Netherlands; ^5^ Center for Proteomics and Metabolomics, Leiden University Medical Center, Leiden, Netherlands; ^6^ Department of Immunohematology Diagnostics, Sanquin, Amsterdam, Netherlands; ^7^ Translational Immunohematology, Research, Amsterdam, Netherlands; ^8^ Department of Hematology, Leiden University Medical Centre, Leiden, Netherlands

**Keywords:** FNAIT, thrombocytopenia, SPRi (surface plasmon resonance imagery), IgG, fucosylation, platelet, FcgRIIIa, alloimmune

## Abstract

Fetal and neonatal alloimmune thrombocytopenia (FNAIT) can occur due to maternal IgG antibodies targeting platelet antigens, causing life-threatening bleeding in the neonate. However, the disease manifests itself in only a fraction of pregnancies, most commonly with anti-HPA-1a antibodies. We found that in particular, the core fucosylation in the IgG-Fc tail is highly variable in anti-HPA-1a IgG, which strongly influences the binding to leukocyte IgG-Fc receptors IIIa/b (FcγRIIIa/b). Currently, gold-standard IgG-glycoanalytics rely on complicated methods (e.g., mass spectrometry (MS)) that are not suited for diagnostic purposes. Our aim was to provide a simplified method to quantify the biological activity of IgG antibodies targeting cells. We developed a cellular surface plasmon resonance imaging (cSPRi) technique based on FcγRIII-binding to IgG-opsonized cells and compared the results with MS. The strength of platelet binding to FcγR was monitored under flow using both WT FcγRIIIa (sensitive to Fc glycosylation status) and mutant FcγRIIIa-N162A (insensitive to Fc glycosylation status). The quality of the anti-HPA-1a glycosylation was monitored as the ratio of binding signals from the WT versus FcγRIIIa-N162A, using glycoengineered recombinant anti-platelet HPA-1a as a standard. The method was validated with 143 plasma samples with anti-HPA-1a antibodies analyzed by MS with known clinical outcomes and tested for validation of the method. The ratio of patient signal from the WT versus FcγRIIIa-N162A correlated with the fucosylation of the HPA-1a antibodies measured by MS (r=-0.52). Significantly, FNAIT disease severity based on Buchanan bleeding score was similarly discriminated against by MS and cSPRi. In conclusion, the use of IgG receptors, in this case, FcγRIIIa, on SPR chips can yield quantitative and qualitative information on platelet-bound anti-HPA-1a antibodies. Using opsonized cells in this manner circumvents the need for purification of specific antibodies and laborious MS analysis to obtain qualitative antibody traits such as IgG fucosylation, for which no clinical test is currently available.

## Introduction

Antibodies of the IgG class are the most abundant isotype in humans and the only antibody type actively transported across the placenta (1). For protection but also immune pathologies, such as autoimmunity and alloantibody-mediated diseases in pregnancy, the quantity of antibodies has a clear relationship with the severity of the disease. However, exceptions exist to this rule. For example, in alloimmunity, this is not always the case (2–4). Recent findings suggest that post-translational modifications in the form of altered glycosylation shape these immune responses.

IgG, like most secreted proteins, is a glycoprotein and contains a single highly conserved *N*-linked glycosylation site on the asparagine residue (N) at position 297. This glycan has a biantennary structure, with a core consisting of mannoses and *N*-acetylglucosamines (GlcNAc) that can be further extended by a bisecting GlcNAc, one or two galactoses, one or two sialic acid residues if galactose is present, and a core fucose. While most IgG responses are fully fucosylated, as exemplified by the fact that total plasma IgG is ~94% fucosylated, some antigen-specific responses can be dominated by afucosylated IgG (5, 6). To date, these antigens stimulating afucosylated IgG seem to be exclusively foreign surface proteins expressed on otherwise normal cells, such as those of enveloped viruses (6), alloantigens [e.g., transplant/HLA antigens, paternal antigens expressed by fetal cells but recognized by the maternal immune system (2, 3, 7–9)], or antigens by pathogens expressed on host cells, (e.g., by the RBC stage of *Plasmodium falciparum* (10) and reviewed in (5). Afucosylated antibodies thus contribute to a more pronounced immune response in both pathologic and beneficial scenarios (5, 11). This notion of improved immune responses is attributed to their enhanced affinity for IgG Fc receptor IIIa (FcγRIIIa; expressed on NK cells, monocytes, and macrophages) and FcγRIIIb (expressed on granulocytes) (12–15). Depending on the cell and the exact trigger, activation of these cells leads to increased phagocytosis and antibody-dependent cellular cytotoxicity. The affinity of FcγRIIIa for IgG with low levels of core fucose at position N297 results in an increase in binding affinity of at least one order of magnitude compared to normally fucosylated antibodies with the same specificity (16). Importantly, this glycan sensitivity is explained by the carbohydrate-carbohydrate interactions at the IgG-Fc N297 - FcγRIIIa-N162 interface (17–19). The appearance of afucosylated IgG responses in alloimmunization during platelet transfusion (8) and also in pregnancy (3, 7, 20) and the enhanced FcγR-mediated functional capacity make this class of responses highly relevant in transfusion medicine and alloimmune responses in pregnancy.

Human platelet antigen (HPA) incompatibility between parents can lead to maternal alloimmunization during pregnancy, resulting in fetal neonatal alloimmune thrombocytopenia (FNAIT). If these HPA-specific antibodies are of the IgG isotype, they are specifically transferred by the neonatal Fc receptor (FcRn) through the placenta and opsonize fetal platelets, resulting in their destruction and hence thrombocytopenia (1, 21, 22). Most cases of FNAIT are caused by HPA-1a-specific antibodies. Due in part to the meager association between anti-HPA-1a antibody levels and the severity of thrombocytopenia, preventive screening is not performed. Rather, in most countries, patient stratification is based on obstetric history, and treatment is only offered to those with a history of severe FNAIT (4). Although in most cases FNAIT is completely asymptomatic or mild, a few cases, when left untreated, can cause severe internal bleeding, with intracranial hemorrhage being the most severe, causing lifelong disability or death (21, 23). High-dose intravenous immunoglobulin (IVIg), which most likely blocks the efficient transport of pathogenic antibodies across the placenta (24), is highly effective, with a success rate of over 98% in preventing intracranial hemorrhage, emphasizing that timely diagnosis is crucial (25).

Effective, quick, and simple glycoanalytics are essential in identifying decreased fucosylation in platelet-directed IgG fractions, which is a common trait observed in the most severe cases of FNAIT. We have previously demonstrated that glycosylation features, when combined with antibody levels, exhibit an association with clinical symptoms. Using a cohort of 166 HPA-1b/b pregnancies from immunized women with HPA-1a positive pregnancies and a history of FNAIT, we purified HPA-1a-specific antibodies and analyzed their IgG glycosylation based on tandem liquid chromatography-mass spectrometry of a tryptic peptide encompassing the N297 IgG glycan (20). Higher antibody levels, along with decreased fucosylation and increased galactosylation, may play a role in the detection of severe cases of FNAIT. Although the level of fucosylation of anti-HPA1a decreased after the first pregnancy, the glycosylation features of these antibodies remained stable during and after the pregnancy itself. Mass spectrometry-based analyses of IgG glycosylation, especially on antigen-specific IgG levels, require technical capabilities beyond the reach of most laboratories and, even then, are very cumbersome (7, 20). They are not suited for the desired quick diagnostics. Current clinical tests based on anti-HPA1a antibody levels are not predictive enough to determine severity and identify those in need of therapy. Therefore, novel techniques that also evaluate qualitative antibody traits are likely to be needed (26).

In our current work, we investigated how the N162A mutant version of the FcγRIIIa-V158 can be utilized in cSPRi measurements to characterize the antibody-mediated platelet-specific immune response in FNAIT. For this purpose, we made use of a cohort previously characterized by mass spectrometry (20). We chose cSPRi to detect IgG-opsonized cells because it captures the antigen-specific IgG and has shown promising results with red blood cells (RBCs) opsonized by FcγRs sensors (27, 28) or using FcγR-expressing cells as detectors (29, 30). For platelets, this brought along two complications: the likely presence of cytophilic and/or anti-HLA antibodies. One is the HLA expression of platelets (because allogeneic, and most likely not clinically relevant, anti-HLA antibodies are also often generated in pregnancy (31)), the other being the expression of FcγRIIa by platelets, which attract cytophilic, non-specific IgG. We addressed these problems in two ways: We used recombinant C-terminally, site-specifically monobiotinylated FcγRs (19) immobilized on an SPR sensor array to detect the binding of fixed platelets sensitized with patient antibodies. These FcγRs wont detect the cytophilic IgG bound to platelets by FcγRIIa as IgGs contain a single binding site to FcγRs (32). We also circumvented the possible contribution of anti-HLA antibodies by removing HLA with chloroquine treatment (33). This allowed for the focused characterization of antibodies specifically directed against platelet antigens, both quantitatively and qualitatively, which, as far as we are aware, had not been achieved prior to this study.

## Methods

### Antibody production, FcγRs generation, biotinylation

An anti-HPA-1a-specific monoclonal IgG1 antibody, B2G1, was generated as previously described (20). Humanized antibody C17 recognizing platelet glycoprotein IIIa (CD61) was cloned from hybridoma by 3’-RACE PCR as previously described (34). FcγRIIIa-Fc fusion constructs were prepared as previously described (19). All recombinant proteins, such as IgG, were expressed in HEK293F cells (Thermo Fisher Scientific, Waltham, USA) cultured in Erlenmeyer flasks (Corning) in 293 Freestyle expression medium (Thermo Fisher Scientific) at 37 °C, 8% CO_2_, and shaking at 125 rpm (35). To produce afucosylated IgG, 0.2mM 2-deoxy-2-fluoro-l-fucose (2FF) (Carbosynth, MD06089) was added to the cells 1 h before transfection (35). Supernatants were collected by centrifugation at 4000g and filtered through 0.45 μm Whatman syringe filters (Merck, Germany). Affinity purification of IgG and FcγRIIIa-Fc fusion proteins was carried out using AKTA Prime (GE Healthcare, Chicago, USA) and a HiTrap Protein A HP column (GE Healthcare). Fractions containing proteins were collected and dialyzed overnight against phosphate-buffered saline (PBS) using Slide-A-Lyzer Dialysis Cassettes, 10kDa molecular weight cutoff (Thermo Fisher Scientific). Liquid chromatography-mass spectrometry analysis of IgG Fc glycosylation of B2G1 glycoforms was performed as previously described (28). FcγRs were site-specifically biotinylated on the BirA tag using the BirA enzyme as previously described (19, 36). 1μM FcγR protein was biotinylated with 0.00657μM BirA ligase. After biotinylation overnight at 25°C, the FcγR sample was buffer exchanged and subsequently concentrated in PBS, pH 7.4, using Amicon Ultra centrifugal filter units (MWCO 30kDa) (Merck). C17 and hIgG1 isotype control (anti-TNP) were biotinylated using EZ-Link Sulfo-NHS-biotin (Thermo Scientific) according to the standard procedure by incubation at room temperature for 2 h at a molar (biotin:antibody) ratio of 20:1. Zebaspin desalting columns (Thermo Fisher Scientific) were used to remove leftover biotinylation reagents.

### Platelet preparation and opsonization

Platelets were isolated from EDTA-anticoagulated blood by centrifugation for 15 min at 210g, fixed with 1% paraformaldehyde for 5 min, then washed twice, and resuspended to a final concentration of 2-5 x 10^8^/mL in sequestrine buffer (17.5 mM Na_2_HPO_4_, 8.9 mM Na_2_EDTA, 154 mM NaCl, pH 6.9) with 0.1% (w/v) bovine serum albumin (BSA). Platelets were HLA-stripped by chloroquine treatment by incubation with 200 mg/ml chloroquine for 1 h at 37°C prior to platelet fixation. Platelets from HPA-1-positive blood donors were used.

FNAIT serum samples, previously characterized in detail (20), were heat-inactivated at 56°C for 30 minutes to prevent complement activation (which would interfere with opsonization and SPR detection), then centrifuged for 10 min at 10000g to remove aggregates, aliquoted, and stored at -20°C. Although plasma samples could also be used, we restricted this study to serum samples. A total of 2 x 10^7^ platelets were added per well to round-bottomed 96-well microtiter plates, washed, and subsequently opsonized with one of the control samples: B2G1 spiked in blood group AB serum (without anti-A or anti-B ABO antibodies; Sanquin, Amsterdam, Netherlands), a negative control sample (AB serum) only, or unknown serum samples that were diluted in PBS + 0.05% BSA and 5 mM EDTA (system buffer), or system buffer only. Opsonization was carried out for 30 min at room temperature with gentle shaking. The platelets were then washed three times by centrifugation at 2000g for 2 min and resuspended in system buffer.

### SPR sensor and measurement

Biotinylated FcγRs, C17, isotype control hIgG1, and anti-IgG nanobodies (Captureselect, Thermo Fisher Scientific) were spotted using a continuous flow microspotter (Wasatch Microfluidics, Schott AG, Mainz, Germany) onto a SensEye G-Streptavidin sensor (Senss BV, Enschede, The Netherlands) at varying densities in triplicates, all diluted in PBS + 0.075%(v/v) Tween-80. Anti-CD16 (Sanquin, clone: 5D2) at 2 μg/ml or Adalimumab (Abbvie, Chicago, USA) at 20 μg/ml was used to detect the spotted FcγRs and their binding capacity (R_max_). The opsonized platelets were injected over the sensor, after which the flow was stopped to allow sedimentation of the RBCs onto the sensor surface for 20 minutes. This was followed by washing at increasing flow speeds (1, 2, 4, 8, 10, 20, 40, 80, and 120 μl/s), by pumping 1 ml of system buffer into the flow chamber. Between the cycles and before measurement, the sensor was regenerated with 20 mM H_3_PO_4 + _0.075%(v/v) Tween-80.

Our strategy to measure the FNAIT samples was to include platelet samples opsonized using the following assays: (1) a standard curve of 0:100, 25:75, 50:50, 75:25, and 100:0 ratios of B2G1+F: B2G1-F at a total concentration of 0.125 μg/ml; (2) two antibody level controls at 0.25 and 0.03125 μg/ml B2G1+F, all spiked in five times diluted Ab+ negative control samples to define the sensitive range; (3) serum samples diluted five and 25 times, respectively, to ensure that at least one of the dilutions falls within the determined concentration range; and (4) a fivefold diluted anti-AB+ negative control sample. A total of 24 measurement cycles were carried out in one measurement, plus buffer controls and hIgG1 controls, to measure the binding capacity of the spotted receptors. All dilutions were prepared in a system buffer.

The response measured at each region of interest on the sensor was expressed in resonance units as a function of time in each cycle as a sensogram. The area under the curve was calculated from each sensogram for comparison. A quadratic polynomial regression fit was applied to determine R_max_ between measurement cycles and calculate the corrected area under the curve (AUC), while a linear regression was fitted to the control platelets opsonized with increasing ratios of B2G1-F as a standard curve as a basis for interpolation. As a readout of the unknown samples, the FcγRIIIa binding index was calculated to indicate the AUC ratio of mutant vs. WT binding, corresponding to the standard curve at 100 - % B2G1^+^F in the control.

### Statistical analysis

The Pearson correlation coefficient was calculated for the comparison of MAIPA- and LC-MS-based methods and the FcγRIIIa binding index, AUC, and two-tailed t-test to compare the ITP and FNAIT cohorts. Results were considered significant at p < 0.05.

### Serum samples and ethics statement

Serum and platelet samples were assigned a specimen number, and no personal identifiers were included in the database. All donors provided informed consent and approved the testing of their samples for research purposes. The detailed description of the FNAIT cohort has been published previously (20) and was classified according to the Buchanan severity score (37). The ITP cohort in this study comprised 49 patient samples from thrombocytopenic individuals sent to our laboratory for platelet autoantibody detection. The mean age of the patients was 45 years (range 2-82), with 27 women and 22 men. Of the 49 patients, 44 had confirmed platelet-specific antibodies detected by either platelet fluorescence tests or MAIPA-based assays. Autoantibody specificities were heterogeneous (beyond the scope of this work), ranging from the recognition of glycoproteins IIb/IIIa, Ib/IX, and V, with many samples containing antibodies to multiple antigens.

## Results

**Figure 1:**
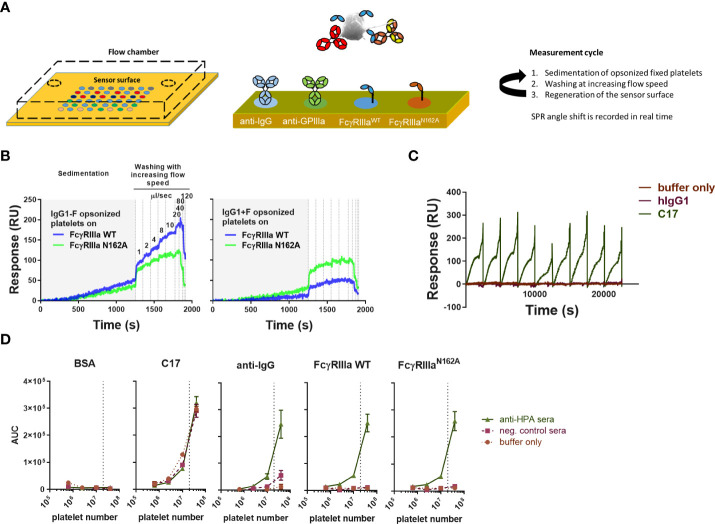
Overview of platelet SPR (surface plasmon resonance) imaging. **(A)** A gel-type streptavidin sensor surface was functionalized with biotinylated ligands, such as anti-human IgG, anti-CD61 (clone: C17), FcγRIII, and FcγRIIIa-N162A. Fixed platelets were opsonized using serum samples or monoclonal, glycoengineered antibodies prior to the measurements, then injected into the flow chamber with the functionalized sensor at the bottom, and their interaction with each ligand was measured in real time. In each measurement cycle, platelets were incubated in the flow chamber without agitation to enable their sedimentation onto the sensor, followed by washing with buffer at increasing flow speeds, pumping 1ml of buffer in each incremental washing step, resulting in a characteristic binding response curve shown in resonance units (RU) [also shown in **(B, C)**]. **(B)** Glycoengineered anti-human platelet antigen 1a (HPA1a) specific IgG1 (clone: B2G1) was generated in fucosylated (normal levels of core fucosylation, IgG1+F) and non-fucosylated (IgG1-F) versions to study their interaction with wild-type FcγRIIIa (FcγRIIIa) and its mutant version, FcγRIIIa-N162A lacking the glycan at amino acid position 162. Each binding response curve was evaluated by calculating the area under the curve (AUC) for comparison. **(C)** Measurements were performed in cycles, regenerating the sensor surface after each platelet sample. CD61-specific platelet interactions are shown in **(B)** throughout the measurement cycles compared to isotype control hIgG1. **(D)** A 5-fold dilution of pooled anti-HPA1a (aHPA) and anti-HPA1a negative AB+ serum (negser) samples was used to opsonize platelet numbers and show their specific interaction with the spotted ligands and lack of non-specific binding to the negative control bovine serum albumin (BSA). All plots are representative of n ≥ 3 independent measurements. The symbols in **(D)** represent the mean values, and the error bars represent the standard deviation of three replicates.

To set up the measurement, we employed a cSPRi configuration where the position of the sensor surface at the bottom of the flow chamber allows for the sedimentation of the cells. The sensors were functionalized by spotting each biotinylated ligand on an area of a 6x8 streptavidin-coated array of 48 spots to detect platelets and platelet-bound antibodies (**Figure 1A**). The chip was equipped with anti-IgG and mutant FcγRIIIa-N162A to detect IgG-opsonized platelets (irrespective of the glycosylation status of the opsonizing antibody). Platelets may carry IgG bound to their FcγRIIa, which would be detected by the anti-IgG spot but not by the mutant Fc FcγRIIIa-N162A. Moreover, human IgG1 C17 (recognizing CD61 and integrin beta 3 in glycoprotein IIb/IIIa) was used to detect platelets independent of IgG (injection control), isotype control, and BSA to determine background signals. Platelets were then injected underflow and allowed to react with the sensor by stopping the flow. This sedimentation was allowed to take place for 1200 seconds before resuming the flow (**Figure 1C**). The flow was incrementally increased to remove weakly bound platelets, a process we have previously described as being in direct relationship to the avidity between the cell and the ligand on the SPR chip (28). C17 and isotype control spots (human IgG1) were monitored during each injection in each run (**Figure 1C**). For high-affinity interactions, e.g., C17, increasing the flow speed to maximum never showed cell release. Presumably, the platelets were bound too tightly and were pressed even more against the sensor underflow. Regeneration with a relatively strong regenerating agent, 20 mM H_3_PO_4 _+_ _0.075%(v/v) Tween-80, enabled multiple measurements using the same sensor (**Figure 1C**).

Platelets opsonized with non-fucosylated and fucosylated glycoforms of B2G1 (anti-HPA-1a) (glycosylation profile shown in **Supplementary Figure 1**) showed, as expected, a differential response on the spots containing FcγRIIIa WT and FcγRIIIa-N162A (**Figure 1B**). While the FcγRIIIa-N162A was not affected by B2G1 fucosylation, the WT variant showed a strongly increased binding to the afucosylated variant, even compared to the FcγRIIIa-N162A, which bound better to fucosylated IgG1 than the WT variant (**Figure 1B**). To evaluate each binding interaction, we calculated the integrated area under the curve (AUC) for each binding interaction. These AUCs showed a clear dependency on the number of platelets injected, with 20 million platelets generally giving clear, strong signals (**Figure 1D**), and were therefore used in all further experiments. All analytes on the sensor provided results with negligible background, except for anti-IgG, where a weak binding was measurable after opsonization with 20% negative AB+ serum (**Figure 1D**). We attributed this to the presence of FcγRIIa on the platelets and therefore decided to compare the binding to FcγRIIIa with the binding response to FcγRIIIa-N162A.

**Figure 2:**
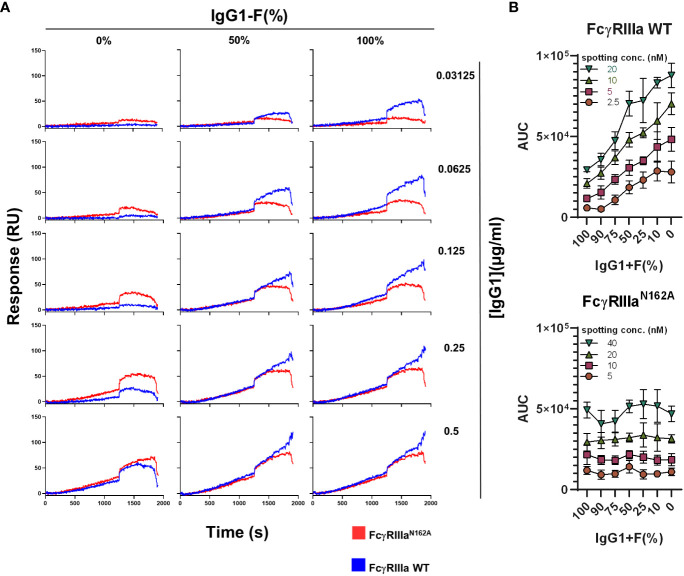
Interaction of FcγRIIIa and FcγRIIIa-N162A with platelets opsonized with fucosylated and non-fucosylated anti-human platelet antigen 1a (HPA1a)-specific monoclonal antibodies (clone: B2G1). **(A)** Representative binding curves of the platelets opsonized with various concentrations of fucosylated and non-fucosylated B2G1 antibodies and their mixtures on the FcγRIIIa (20nM) and FcγRIIIa-N162A (40 nM) spots. **(B)** Binding of platelets opsonized with 0.125 µg/ml B2G1 with various ratios of non-fucosylated and fucosylated antibodies to FcγRIIIa (top) and FcγRIIIa-N162A (bottom), expressed as AUC of the binding curve at various spotted ligand densities, is shown. Representative plots of n ≥ 3 independent measurements. The symbols represent the mean values, and the error bars represent the standard deviation of four replicates.

We then investigated the sensitivity to IgG opsonization of platelets and the level of fucosylation in the signal generation of FcγRIIIa-WT and FcγRIIIa-N162A. Irrespective of the IgG concentration used, FcγRIIIa-N162A on the array was not affected by the fucosylation status of the antibodies, whereas FcγRIIIa-WT was. Higher concentrations gave stronger signals, while lower concentrations allowed for better discrimination based on the level of fucosylation (**Figure 2A**). These results demonstrate that the presence of the afucosylated B2G1 strongly affects the binding reaction on FcγRIIIa-WT and enables binding even at very low antibody concentrations, where normally fucosylated antibodies do not give any response. When expressed as AUC, a similar sensitivity of only FcγRIIIa-WT was seen as a function of the level of fucosylation, regardless of the amount of FcγR spotted (**Figure 2B**). A slightly different spotting concentration range of FcγRIIIa-WT and FcγRIIIa-N162A was necessary to ensure a comparable range of binding capacity for the two receptors, which is explained below.

**Figure 3:**
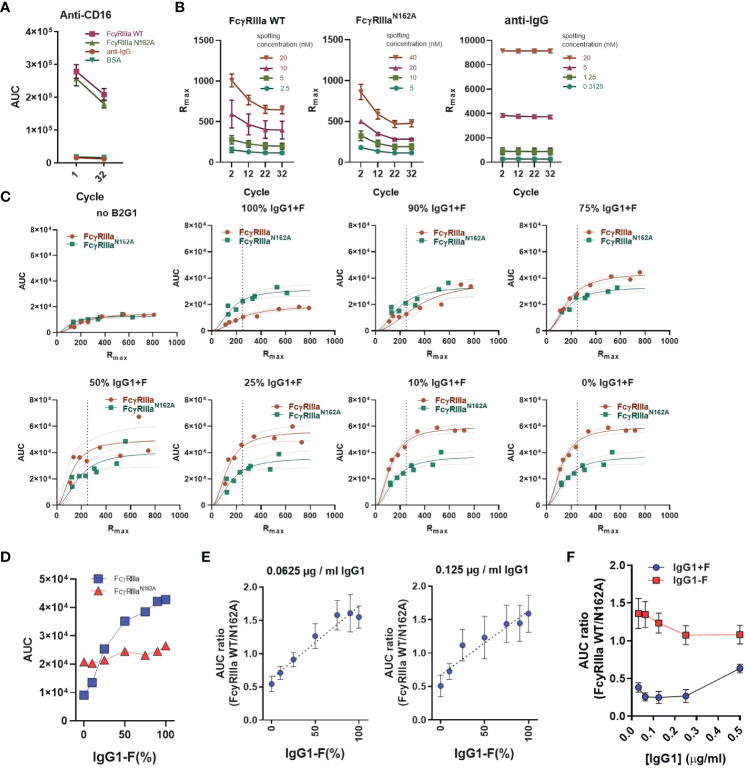
AUC ratio of FcγRIIIa and FcγRIIIa-N162A after B2G1 glycosylation. **(A)** To determine the presence and stability of the spotted FcγRIIIa anti-CD16 antibody (5D2 at 2 µg/ml) injected at the beginning and end of the measurement, BSA and anti-human IgG (aIgG) are shown as negative controls. **(B)** To determine the binding capacity of the spotted ligands, hIgG1 (adalimumab) at 10 mg/ml was injected at regular intervals throughout the measurements, and the maximum of the measured response (R_max_) was used to characterize each spotted ligand. **(C)** Instead of spotting concentration, the area under the binding response curve (AUC) is presented as a function of R_max_ to compare the binding of opsonized platelets to FcγRIIIa and FcγRIIIa-N162A. A quadratic polynomial regression was fitted to calculate the corrected AUC value at any given R_max_. **(D)** The corrected AUC values at R_max_ = 250 of the plots shown in **(C)**. **(E)** The calculated AUC ratio of the binding response to FcγRIIIa and FcγRIIIa-N162A after opsonization of platelets (2*10^7^) with 0.125 and 0.0625 µg/ml IgG1 (B2G1) mixtures containing fucosylated and non-fucosylated antibodies as indicated, diluted in system buffer containing 4% negative control serum. This B2G1 standard curve was later used to determine the binding activity to FcγRIIIa WT of the unknown serum samples based on interpolation using the fitted linear regression equation. **(F)** AUC ratios of platelets (2*10^7^) opsonized with fucosylated (IgG1+F) or non-fucosylated B2G1 IgG1 (IgG1-F) at concentrations ranging from 0.03125 to 0.5µg/ml B2G1. In **(E)**, aggregated values of four independent measurements are shown, whereas **(A–C, F)** are representative of n ≥ 3 independent measurements. The symbols in **(A, B, E, F)** represent the mean values, and the error bars show the standard deviation of the triplicates. In **(C)**, individual measurement points are shown with ribbons representing the 95% confidence interval of the fitted equation.

The reactivity of the spots, especially the FcγR spots, was evaluated at the start and end of multiple injections with anti-FcγRIII antibodies and a slight drop in reactivity was noticed (**Figure 3A**). To evaluate this using less avid interactions (requiring stronger regeneration), we determined the binding capacity of the FcγR and the anti-IgG spots with monomeric IgG1 at 20 μg/ml at every 10^th^ cycle. With this approach, we characterized each ROI with a maximum response (R_max_) and used this value as the basis for comparing the functional binding capacity across all runs (**Figure 3B**). In each cycle, each sample was measured on multiple spots on the array, each containing a different active concentration (**Figure 3C**), with each active concentration interpolated based on the cycle in the run (**Figure 3B**). This allowed us to compare the measured AUC with the interpolated R_max_ for each cycle. Depending on the relative amount of fucosylated IgG1 opsonizing the platelets (B2G1), the response for the FcγRIIIa-WT receptor gradually increased, whereas the response for the mutant remained stable. We chose an R_max_ of 250 as a basis for comparison to evaluate our measurements (**Figure 3D**) and calculated the AUC ratio measured on the receptors to simplify the readout. Our goal here was to obtain a single indicator that would provide information on the difference in antibody binding activity to FcγRIIIa due to changes in glycosylation. Using 0.0625 and 0.125 μg/ml B2G1-F and B2G1+F, covering a range of fucosylation from 1 to 100%, the AUC ratio-based standard curves remained stable (**Figure 3E**). Therefore, in each measurement with the unknown serum samples, we included these standard curves in the measurement and used them as calibrators by fitting a linear regression and interpolating the binding activity of the unknown serum samples. Since this approach did not allow us to draw conclusions about the exact glycosylation status of the unknown samples, we introduced a FcγRIIIa binding index as a readout of the AUC ratios, where FcγRIIIa binding index indicates stronger binding activity to the receptor corresponding to the calculated value at 100% B2G1+F based on the standard curve. Importantly, this ratio was found to be reliably stable only at lower opsonization levels, below 0.25 μg/ml, since at high opsonization levels, the avidity presumably compensates for the lower affinity, and the AUC values converge to 1. (**Figure 3F**). These calibration steps serve as a basis for comparison to characterize the binding profile of platelets opsonized with unknown serum samples.

**Figure 4:**
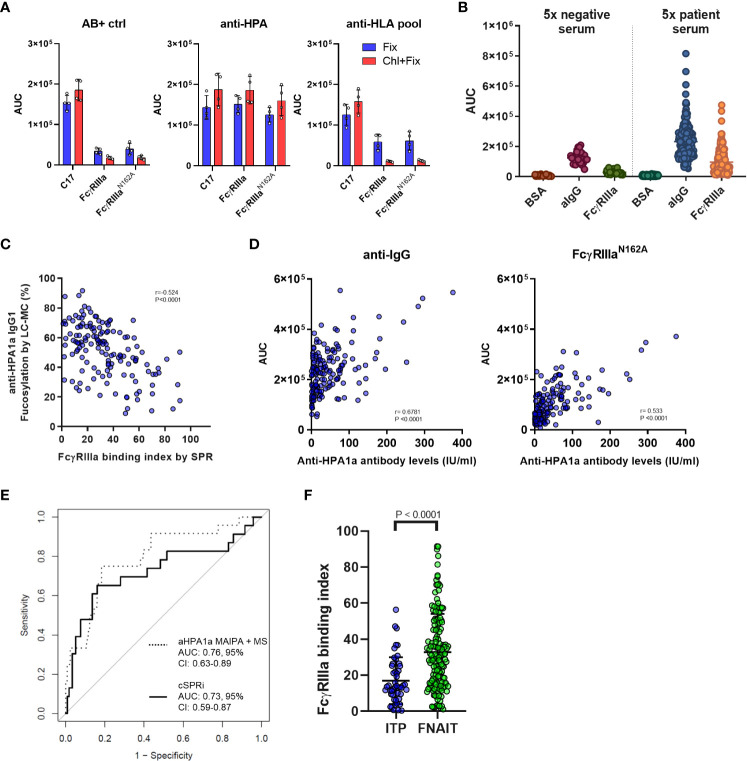
Platelet cellular SPR imaging-based evaluation of fetal neonatal alloimmune thrombocytopenia (FNAIT) and immune thrombocytopenia (ITP) serum samples. **(A)** Reactivity of serum samples with chloroquine-treated and fixed platelets only is compared following opsonization with 20% anti–HPA serum, anti-HLA serum pool, or control negative AB+ serum samples. AUC values determined with FcγRIIIa (20 nM spotting concentration) and FcγRIIIa-N162A (20 nM) and anti-CD61 (C17, 10 nM) antibodies are shown. The symbols represent individual replicates of two independent measurements, and the error bars show the standard deviation. **(B)** Binding of platelets opsonized with serum samples from the FNAIT cohort to anti-IgG negative control BSA and FcγRIIIa WT. Each symbol represents platelets opsonized with a fivefold diluted serum sample. n=143 FNAIT samples and n=28 negative controls are shown. **(C)** Correlation scatter plots of anti-HPA1a fucosylation and galactosylation as measured by mass spectrometry with the determined FcγRIIIa binding index based on standard curves determined in the same measurement for each serum sample. A significant negative correlation was found for fucosylation and a weak positive interaction for galactosylation. **(D)** Correlation scatter plots of anti-HPA1a titer as determined by MAIPA and the binding determined on the FcγRIIIa-N162A and anti-human IgG(aIgG) spots. In **(C)** and **(D)**, Pearson correlation coefficients (r) were calculated and were considered statistically significant at p < 0.05. **(E)** Receiver operating characteristic curve-based comparison of mass spectrometry-based glycan profiles and MAIPA-based antibody levels; and platelet SPR imaging-derived FcγRIIIa binding index and binding response to FcγRIIIa-N162A-based identification of severe disease cases (Buchanan score > 2). **(F)** Comparison of the FcγRIIIa binding index measured in fixed platelet samples from patients with immune thrombocytopenia (ITP, n = 49) and results from the FNAIT cohort. The statistical analysis was performed using a two-tailed t-test.

Since the unknown samples may also contain anti-HLA antibodies, we decided to measure the FNAIT samples by opsonizing chloroquine-treated platelets, thereby denaturing the platelet HLA (33). Fixed platelets were opsonized with anti-AB+ negative control serum, pooled anti-HPA-1a FNAIT serum, or pooled anti-HLA serum. Our results showed that the chloroquine treatment was effective, as no anti-HLA was detected by any of the FcγRs on the chip, while in the case of anti-HPA serum samples, the binding remained comparable (**Figure 4A**). Therefore, chloroquine-treated platelets were used for the evaluation of 166 FNAIT samples. In total, 23 samples were excluded due to either low available sample volumes or a lack of binding in cSPRi. The binding of negative control sera and patient sera to BSA control spots was minimal. Anti-IgG showed background binding, likely due to the binding of platelet FcγRIIa being at least partly occupied by cytophilic irrelevant IgG, as no signal was seen on FcγRs with negative control sera. FNAIT samples showed strong but variable binding to both FcγRIIIa and anti-IgG spots (**Figure 4B**). Next, we looked at the correlation between IgG glycosylation features measured by gold-standard LC-MS, and FcγRIIIa binding index measured by SPR for each FNAIT sample, using Pearson correlation to evaluate their interaction. The LC-MS analysis of anti-HPA-1a Fc-fucosylation showed a moderately strong negative correlation with the FcγRIIIa binding index (**Figure 4C**). In contrast, Fc-galactosylation, -sialylation, and -bisection all showed significant but weak correlations. (**Supplementary Figure 2**).

The anti-platelet immune response may depend on the antibody titer and the strength of FcγR binding. Thus, we tested how well the anti-IgG and FcγRIIIa-N162A spots could be utilized to quantify anti-HPA-1a IgG levels by comparing them with classical Monoclonal Antibody-specific Immobilization of Platelet Antigen (MAIPA) assays, which are standard for anti-platelet antibody quantification (38, 39). We found that FcγRIIIa-N162A showed a superior correlation with anti-HPA-1a IgG levels based on MAIPA as compared to anti-IgG, likely due to the presence of cytophilic antibodies bound to platelet FcγRII (**Figure 4D**). Finally, we examined how the combination of FcγRIIIa-N162A together with the FcγRIIIa binding index explained disease severity compared to the previously established LC-MS-based glycosylation features combined with the MAIPA-based anti-HPA-1a IgG1 levels. The receiver-operator characteristic curves showed comparable results for the outcome, with an AUC of 0.73 (95% CI, 0.59-0.87) for cSPRi and 0.76 (95% CI, 0.63-0.89) for LC-MS (**Figure 4E**), where an AUC of 1.00 indicates perfect and 0.50 random discrimination. We analyzed an ITP cohort, which mostly consisted of adults suspected of having ITP, using a biosensor. The majority of the cohort tested positive for either the platelet immunofluorescence test or MAIPA. We evaluated the FcγRIIIa binding index in a subset of suspected ITP patients with FcγRIIIa binding reactivity and found it to be significantly lower compared to the FcγRIIIa binding index of the FNAIT cohort, suggesting that the role may be less relevant in ITP than in FNAIT (**Figure 4F**). Altogether, these results show that the cellular SPR platform loaded with FcγRIIIa is a feasible method for characterizing platelet antigen-specific antibodies.

## Discussion

General antibody responses result in fucosylated IgG responses (5, 6). IgG responses to platelet and RBC surface antigens are highly variable, ranging from 10-99% fucosylation, with decreasing fucosylation associated with increasing disease severity, likely due to the activity of FcγRIII on immune cells (2, 3, 7, 20). Despite its clinical relevance, quantification of this important trait, especially of the antigen-specific fraction, is not an easy task. The most successful methods involve purification of the antigen-specific fraction and tandem analysis of IgG-derived glycopeptides by liquid chromatography-mass spectrometry, which is not readily adaptable to routine diagnostics. Here, we have devised a simplified method to characterize IgG fucosylation of anti-platelet antibodies using cellular SPR. The results obtained give insight into biological relevance and are on par with the direct measurement of fucosylation as seen by LC-MS.

The pathophysiology of FNAIT is complex and involves multiple possible mechanisms (40) that, in addition to causing platelet destruction through IgG-Fc receptors, may also be affected by complement activation (41) and CRP (42). Platelet production can also be affected by the immune response targeting megakaryocytes (43), and the antibodies can also cause vascular damage by binding to HPA antigens, which are also expressed on endothelial cells (44) and trophoblast cells (45). Moreover, anti-HLA antibodies may play a possible accessory role (which seems to be minor) in combination with anti-HPA antibodies (46, 47). Nevertheless, the available data seem to suggest a strong role for FcγRs that is influenced by IgG glycosylation, which can be fundamentally different for IgG targeting alloantigens, including HPA antigens and in some cases also anti-HLA IgG (8, 9). These changes in IgG glycosylation consist first and foremost of afucosylated IgG that has up to 40-fold elevated affinity for FcγRIII, which may have an even greater impact on functional activities through even stronger avidity effects (14, 48). The effect of IgG fucosylation is restricted to FcγRIII due to a conserved glycan at N162 in this family of receptors, which is not found in human FcγRI and FcγRII. This N162 glycan can only be sterically accommodated in the IgG-Fc in the absence of the core fucose (17, 19, 49). Interestingly, marked differences in the glycosylation of FcγRs *in vivo* have recently been reported (50, 51), which have a slight modifying effect on the affinity towards IgG glycoforms (51, 52).

Here, we estimated the IgG fucosylation based on the binding ratio to FcγRIIIa-V158 and its mutant variant, FcγRIIIa-V158 N162A, which lacks the N162 glycan and binds IgG in a manner insensitive to its fucosylation state. We showed that a biosensor equipped with these receptors captured IgG-opsonized platelets in a concentration-dependent manner. This allowed for the quantification of IgG, which gave results similar to those obtained by MAIPA. As WT FcγRIIIa was additionally sensitive to IgG fucosylation, we used the differential response measured for the N162A variant as a proxy for fucosylation. We and others have previously found that antibody quantity alone is not a good predictor of severity in FNAIT, but adding glycosylation trait information gives a more complete picture, with IgG fucosylation giving the best prediction, followed by IgG galactosylation and quantity (20). The effect of galactose may be because its presence further enhances the binding affinity of afucosylated IgG to FcγRIII (by a factor of 2) (14), or because of its role in enhancing IgG hexamerization and thereby complement activation (53, 54). IgG galactosylation also seems to affect platelet-mediated clearance *in vivo* (8, 41), but the relative importance of these two mechanistic effects of IgG galactosylation on either FcγR or complement is as of yet unknown.

The new simplified method of probing IgG characteristics by cellular SPR also gave an opportunity to test the IgG features of antigen-specific IgG targeting platelets in ITP patients. As of yet, IgG glycan features have not yet been analyzed due to challenges in quantity and specificity, especially because all platelets also carry IgG bound to platelet FcγRIIa. Using the SPR setup allowed us to specifically investigate antigen-bound IgG, as only IgG with a free Fc region, not bound to FcγRIIa, could be detected. Although alloantigens seem to provide the immunological cues required to induce afucosylated IgG (5, 6), it is not yet known whether platelet autoantigens also induce this atypical afucosylated IgG response. Although the results provided herein suggest that the afucosylated features of autoantibodies in ITP are less strongly afucosylated than in FNAIT, they clearly show that the binding quality to FcγRIIIa is variable, indicating that some ITP patients may have afucosylated autoantibodies.

Clearly, more research is needed to better comprehend the pathophysiology of FNAIT and to delineate the role of HPA1a-specific IgG antibodies and their fucosylation therein. The retrospective nature of the cohort used in our study needs to be expanded to a prospective study, which is logistically extremely difficult to realize due to the frequency of this disease and the lack of centralized diagnostics. Similarly, the importance of fucosylation is to be studied further in other areas of hematology, but also in infectious diseases, where allo- or RBC-stage plasmodium-specific afucosylated IgG antibodies play a role in pathogenesis or protection (3, 10, 20). The FcγRIII-based antibody quality profile should be even easier to realize with antibodies against RBCs, as these behave robustly in SPR (27, 28, 55). Comparing the cellular SPR method with FcγRIIIa, the presented approach undoubtedly represents a streamlining step in comparison to the MS-based one, where particular antibodies must be isolated, quantified, and subjected to proteolytic cleavage, LC-MS methods, and then computational glycoanalysis. While the cellular SPR-based technique allows for the immediate evaluation of platelets captured by Fc receptors, the LC-MS-based approach requires weeks at best but also provides information on other glycan features such as galactosylation, which may also affect FcγR binding (although less than afucosylation) and complement activity (14, 53). The preparation of platelet samples for the cellular SPR approach is no different from the methods presently being employed in standard thrombocyte diagnostic laboratories, and therefore doesnt require new protocols for sample preparation. Moreover, the measurements are not limited to thrombocytes alone but encompass the application of RBCs, fixed cells, and live cells. However, it is important to note that the preparation of the sensor, the execution of the measurements for both methods, and their subsequent evaluation remain complicated and require skilled personnel.

Cellular SPR allows for the characterization of cellular surface markers (55, 56), but also for the analysis of opsonized blood cells interacting with receptors of interest (42). Interaction of the opsonized cells only efficiently takes place after the initial sedimentation of the cells by stopping the flow after cell injection (56). Incremental increases in flow speed enable differential quantitative analysis of the avidity of the analyte-ligand interactions (28, 56). We chose to represent this binding in the AUC. However, AUC only gives a rough estimate of the actual binding. We found that AUC ratios, based on the intrinsic affinities of soluble, well-characterized IgG, are in agreement with the expected avidity between opsonized platelets and receptors on the sensor surface. Importantly, we found that low levels of opsonization were necessary to reveal these differences, as platelets opsonized with saturating levels of antibody lost discriminatory power. Also, as we have previously described, washing at an incremental flow speed was necessary to reveal the differences because sedimentation only reflects the stochastic distribution of the cells on the sensor surface and provides very limited information about binding. When washing starts, the opsonized platelets are presumably pressed onto the sensor, enabling more FcγR-opsonized platelet interactions and thus increasing the measured response up to the point where the flow speed is high enough to wash the weakly bound platelets off the sensor. However, the FcγRIIIa binding index provides an indirect evaluation of the biological activity of the antibodies. A limitation of this study is that FcγRIIIa only binds IgG1 and IgG3, and would potentially miss IgG2 and IgG4. However, these responses are likely to be less frequent as IgG1 and IgG3 are the major isotypes seen in anti-HPA-1a responses (57). Opsonized platelets end up sequestered in the liver and/or spleen, and these organs are considered to be the major organs responsible for the clearance of platelets, which mostly contain monocytes and macrophages expressing FcγRIIIa (58–60). While recent results from us and others emphasize the role of FcγRIa in platelet phagocytosis by monocytes and macrophages without FcγRIIIa, they also suggest an at least equally important role for FcγRIIIa in myeloid cells that do express this receptor (60–63). Importantly, this balance is likely to shift towards FcγRIIIa when patients have afucosylated anti-platelet IgG (3, 48). Our results also indicate that a similar FcγRIIIa-based approach could be adopted for flow cytometry. Taken together, our results confirm that FcγRs are in many ways superior to anti-IgG antibodies, not only because they unravel the biological response but also because their application enable a lower background on cases where the investigated cells themselves possess FcγRs (such as platelet FcγRIIa), and therefore may carry cytophilic antibodies.. The potential benefits of this approach using recombinant FcγRs have already been appreciated in recent works from multiple groups (64–66), in particular the use of the FcγRIIIa receptor to characterize IgG fucosylation (67). Here, we have expanded this repertoire to include the detection and characterization of platelet-specific allo- and autoantibodies. Overall, the results suggest that this approach may be generally applicable to determining the biological activity of cell-bound antibodies in other diseases.

## Data availability statement

The raw data supporting the conclusions of this article will be made available by the authors, without undue reservation.

## Ethics statement

The studies involving human participants were reviewed and approved by Sanquin Ethical Advisory Board. The studies were conducted in accordance with the local legislation and institutional requirements. Written informed consent for participation in this study was provided by the participants’ legal guardians/next of kin.

## Author contributions

ZS and AB performed and analyzed SPR experiments; ZS, AB, MS, and DS analyzed the data; ZS, AT, RV, SL- T, JM, and WE provided reagents; EG and MW performed LC-MS experiments and analysis; LP provided platelet samples; ZS and GV wrote the manuscript, which was edited by all authors; LP, CS, MH, and GV supervised the work. All authors contributed to the article and approved the submitted version.
