# Supplementary material for: Cellular surface plasmon resonance-based detection of anti-HPA-1a antibody glycosylation in fetal and neonatal alloimmune thrombocytopenia
Source: Front Immunol. 2023 Oct 5;14:1225603. doi: 10.3389/fimmu.2023.1225603 (PMC10585714; doi:10.3389/fimmu.2023.1225603)
Supplement: Supplementary file 1 [file DataSheet_1.pdf]

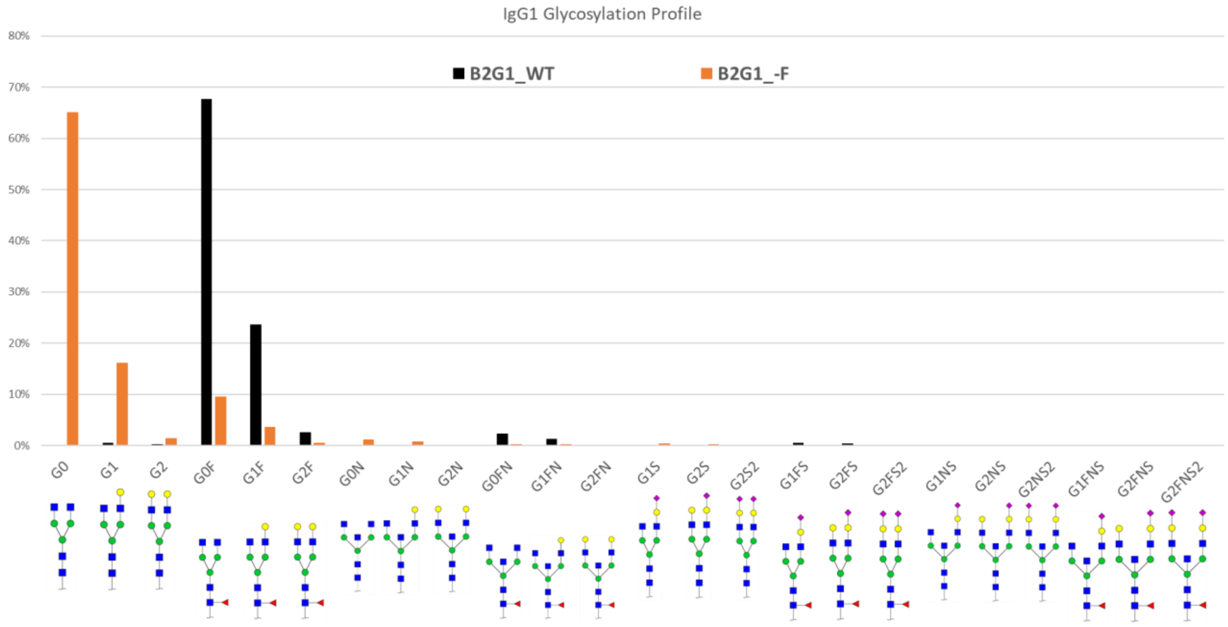

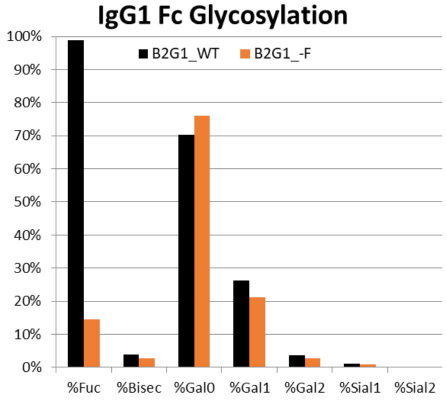

Supplementary Figure 1. B2G1 Fc glycosylation profile

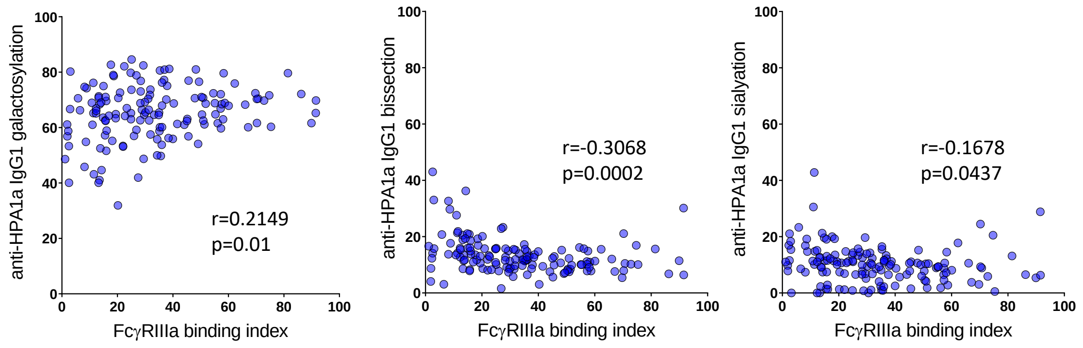

Supplementary Figure 2. Correlation scatterplots of FBI and glycosylation features of anti-HPA1a IgG1 the correlation between IgG glycosylation features, previously measured by LC-MS, and the measured FcγRIIIa binding index by SPR for each FNAIT sample, using Pearson correlation to evaluate their interaction.
